# Annealing Effect on (FAPbI_3_)_1−x_(MAPbBr_3_)_x_ Perovskite Films in Inverted-Type Perovskite Solar Cells

**DOI:** 10.3390/ma9090747

**Published:** 2016-09-03

**Authors:** Lung-Chien Chen, Jia-Ren Wu, Zhong-Liang Tseng, Cheng-Chiang Chen, Sheng Hsiung Chang, Jun-Kai Huang, King-Lien Lee, Hsin-Ming Cheng

**Affiliations:** 1 Department of Electro-Optical Engineering, National Taipei University of Technology, Chung-Hsiao E. Road, Taipei 10608, Taiwan; t9659012@ntut.org.tw (J.-R.W.); tw78787788@yahoo.com.tw (Z.-L.T.); t103658032@ntut.edu.tw (J.-K.H.); kllee@ntut.edu.tw (K.-L.L.); 2 Research Center for New Generation Photovoltaics, National Central University, Taoyuan 32001, Taiwan; chiang0414@ncu.edu.tw (C.-C.C.); shchang@ncu.edu.tw (S.H.C.); 3 Material and Chemical Research Laboratories, Industrial Technology Research Institute, Hsinchu 31040, Taiwan; smcheng@itri.org.tw

**Keywords:** MAPbI_3_, FAI, organic solar cells, perovskite

## Abstract

This study determines the effects of annealing treatment on the structure and the optical and electronic behaviors of the mixed (FAPbI_3_)_1−x_(MAPbBr_3_)_x_ perovskite system. The experimental results reveal that (FAPbI_3_)_1−x_(MAPbBr_3_)_x_ (x ~ 0.2) is an effective light-absorbing material for use in inverted planar perovskite solar cells owing to its large absorbance and tunable band gap. Therefore, good band-matching between the (FAPbI_3_)_1−x_(MAPbBr_3_)_x_ and C_60_ in photovoltaic devices can be controlled by annealing at various temperatures. Accordingly, an inverted mixed perovskite solar cell with a record efficiency of 12.0% under AM1.5G irradiation is realized.

## 1. Introduction

Organic/inorganic hybrid perovskite materials of the form APbX_3_ (A = CH_3_NH_3_ or HC(NH_2_)_2_; X = I, Br or Cl) are attracting considerable interest owing to their potential for absorbing light in solar cells due to their broad spectral absorption, strong light harvesting, and long exciton diffusion length [1,2,3,4,5,6]. Perovskite solar cells (PSCs), which include perovskite materials as active absorption layers, have yielded good device performance with the highest power conversion efficiencies (PCEs) that have risen from approximately η = 3% efficiencies [7] to almost 20.2% [8]. Additionally, perovskite thin films of the form MAPbI_3_ can be prepared by the vapor-assisted solution method [9], the sequential deposition process [10,11,12], the one-step spin-coating method [13] and the two-step sequential method [14]; the corresponding processes are fast, simple, and low-cost. PSCs have been developed with two types of device architecture—conventional [10,11,12,13,14] and inverted [15]. Recently, Jeon et al. [16] reported on the compositional engineering of high-performance PSCs. They used mixed CH_3_NH_3_PbBr_3_ (MAPbBr_3_) and HC(NH_2_)_2_PbI_3_ (FAPbI_3_) in a solvent as a precursor to deposit (FAPbI_3_)_x_(MAPbBr_3_)_1−x_ perovskite films on mesoporous TiO_2_ to form conventional PSCs. However, to the best of our knowledge, no detailed study of the mixing of FAPbI_3_ and MAPbBr_3_ perovskite in inverted PSCs has been published.

This work reports solution-process (FAPbI_3_)_1−x_(MAPbBr_3_)_x_ perovskites (that are formed using a mixed solution of HC(NH_2_)_2_PbI_3_ (FAPbI_3_) and CH_3_NH_3_PbBr_3_ (MAPbBr_3_)) for use in PSCs with an inverted architecture. The (FAPbI_3_)_1−x_(MAPbBr_3_)_x_ perovskites are formed by a one-step spin-coating process on PEDOT:PSS-coated ITO substrates. The optical, structural, and surface properties of the (FAPbI_3_)_1−x_(MAPbBr_3_)_x_ perovskite films are investigated as functions of the thermal annealing temperature; the relationship between the performance of the PSC and the properties of the perovskite films is discussed.

## 2. Methods

**Figure 1:**
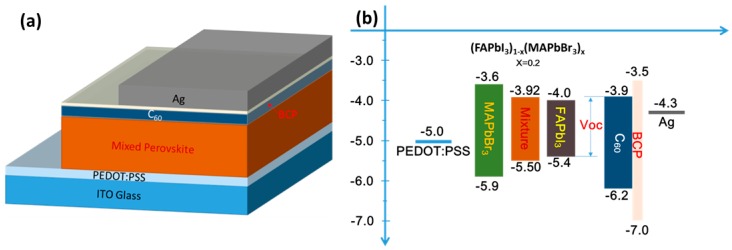
(**a**) Schematically depicts the complete structure and (**b**) corresponding energy band diagram in this study.

In this study, a PEDOT:PSS film using a Trition X-100 (volume ratio PEDOT:PSS:Trition X-100 = 150:1) surfactant was spin-coated on a pre-cleaned ITO substrate at 5000 rpm for 30 s. After spin coating, the film was annealed at 140 °C for 10 min. The perovskite layer was deposited using the solvent-engineering technique [13] using a mixing solvent (in which the volume ratio of dimethyl sulfoxide (DMSO) to γ-butyrolactone (GBL) was 1:1). Then 0.96 mmol of FAPbI_3_ and 0.24 of mmol MAPbBr_3_ (i.e., 165 mg of FAI, 27 mg of MAB, 443 mg of PbI_2_ and 88 mg of PbBr_2_) were dissolved in the mixing solvent (1 mL) as a (FAPbI3)_0.8_(MAPbBr3)_0.2_ precursor solution. The perovskite precursor solution was coated onto the PEDOT:PSS/ITO substrate in two consecutive spin-coating steps at 1000 rpm and 5000 rpm for 10 s and 20 s, respectively. At 5000 rpm, the wet spinning film was quenched by dropping 50 μL of anhydrous toluene onto it. After spin coating, the film was annealed at 100 °C for 10 min. Subsequently, C_60_, Bathocuproine (BCP), and a silver (Ag) electrode were deposited to thicknesses of 50, 5 and 100 nm, respectively, using a thermal evaporator. The sample was covered with a shadow mask to define an active area of 0.5 cm × 0.2 cm during C_60_/BCP/Ag deposition. Figure 1a schematically depicts the complete structure.

### Material and Device Measurement

The crystalline microstructures of the films were determined using a PANalytical X’Pert Pro DY2840 X-ray diffractometer with Cu-Kα radiation (PANalytical, Naerum, Denmark) (λ = 0.1541 nm). A field-emission scanning electron microscope (GeminiSEM, ZEISS, Oberkochen, Germany) was used to observe the surface morphology of the cells. Photoluminescence (PL) and absorption spectra were measured using a fluorescence spectrophotometer (Hitachi F-7000) (Hitachi High-Technologies Co., Tokyo, Japan) and a UV/VIS/NIR spectrophotometer (Hitachi U-4100 spectrometers) (Hitachi High-Technologies Co., Tokyo, Japan), respectively. Moreover, the current density-voltage (J-V) characteristics were measured using a Keithley 2420 programmable source meter (Keithley, Cleveland, OH, USA) under irradiation by a 1000 W xenon lamp. The scan rate is 0.1 V/s. Finally, the irradiation power density on the surface of the each sample was calibrated as 1000 W/m^2^.

## 3. Results and Discussion

**Figure 2:**
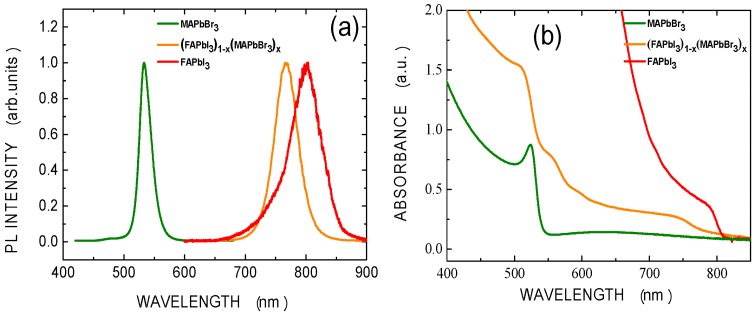
(**a**) PL and (**b**) absorption spectra of MAPbBr_3_, (FAPbI_3_)_1−x_(MAPbBr_3_)_x_, and FAPbI_3_ films deposited on glass substrates.

Mixed perovskite can be flexibly modified by changing the concentration ratio of the mixed halogen precursors [16,17]; this process is useful for photovoltaic applications. The LUMOs of FAPbI_3_, MAPbBr_3_ and C_60_ are −4.0, −3.6 and −3.9 eV, respectively [18,19]. To optimize the band matching with C_60_, the mole ratio of FAPbI_3_ to MAPbBr_3_ in the mixed perovskite was set to 80:20 (i.e., x = 0.2) because the LUMO of the (FAPbI_3_)_0.8_(MAPbBr_3_)_0.2_ was determined by interpolation to be −3.92, as presented in Figure 1b. Figure 2a displays the PL spectra of the MAPbBr_3_, (FAPbI_3_)_1−x_(MAPbBr_3_)_x_, and FAPbI_3_ films with annealing treatment at 135 °C. The spectra include peaks at 530, 762 and 800 nm, respectively. The ratio of the concentration of the (FAPbI_3_)_1−x_(MAPbBr_3_)_x_ film, x, is estimated to be around 0.2 which is similar to the mole ratio in the precursor solution. The absorption edges of the three perovskite films are clearly shifted, as shown in Figure 2b.

**Figure 3:**
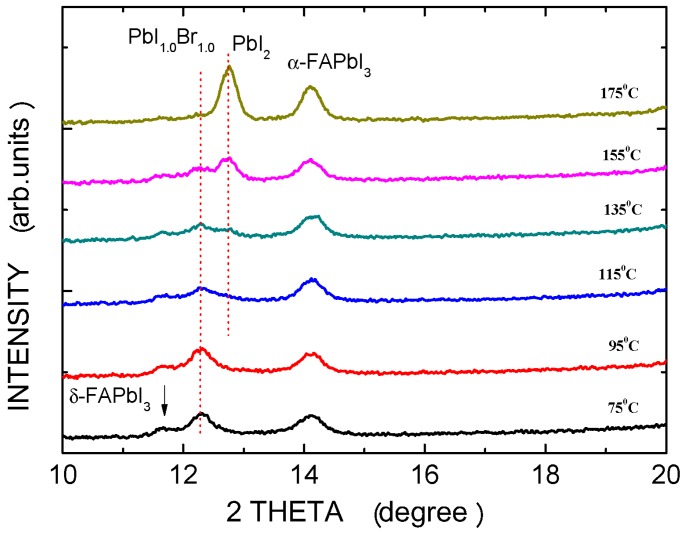
XRD patterns of (FAPbI_3_)_0.8_(MAPbBr_3_)_0.2_ perovskite films following annealing at various temperatures.

Figure 3 shows the XRD patterns of (FAPbI_3_)_1−x_(MAPbBr_3_)_x_ perovskite films after thermal annealing at various temperatures. The spectra include four main diffraction peaks at 11.67°, 12.28°, 12.71°, and 14.11°, which correspond to the δ-FAPbI_3_, PbI_1.0_Br_1.0_, PbI_2_, and α-FAPbI_3_ phases, respectively. As the annealing temperature increases, the intensity of the α-FAPbI_3_ phase peak increases and that of the δ-FAPbI_3_ phase peak decreases because δ-FAPbI_3_ transitions to the α-FAPbI_3_ phase or the crystallinity of the α-FAPbI_3_ phase is increased. Annealing at 175 °C completely suppresses the formation of the δ-FAPbI_3_ phase, suggesting that perovskite films must be thermally annealed to drive the inter-diffusion of precursors [20]. The δ-FAPbI_3_ powders can also be converted to α-FAPbI_3_ powders by annealing and this process is reversible even after the δ-FAPbI_3_ powders have been stored in air for 10 h [16]. The peak intensity of the PbI_1.0_Br_1.0_ phase decreased as the annealing temperature increased, vanishing at 175 °C, whereas the peak intensity of the PbI_2_ phase increased with an annealing temperature over 135 °C, perhaps because methylammonium bromide (MABr) and methylammonium iodide (MAI) were thermally decomposed from the films, forming the PbI_2_ phase. Furthermore, the intensity of the α-FAPbI_3_ phase peak increased with the annealing temperature owing to the stability of formamidinium iodide (FAI) at high temperatures.

**Figure 4:**
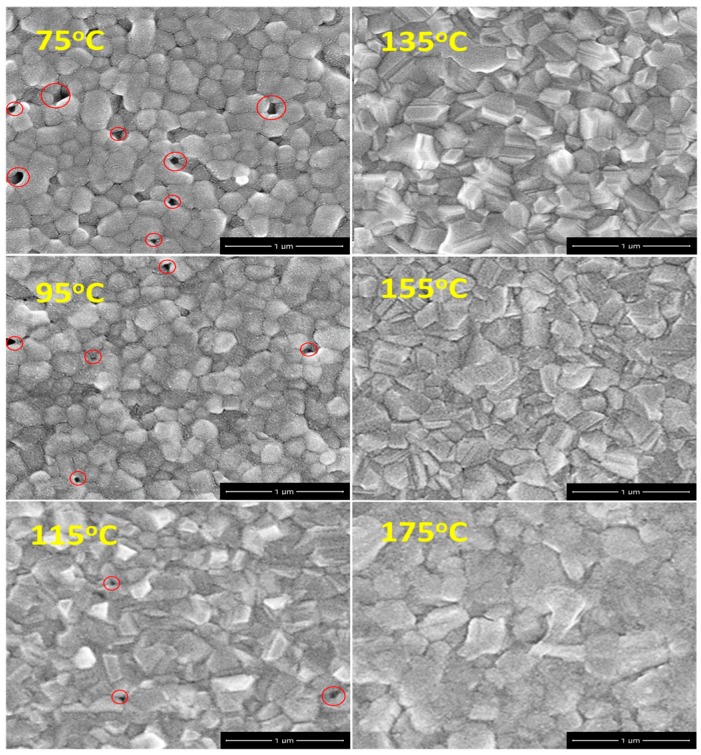
SEM images of (FAPbI_3_)_0.8_(MAPbBr_3_)_0.2_ perovskite films annealed at various temperatures. Red circles indicate defect sites.

Figure 4 presents SEM images of (FAPbI_3_)_1−x_(MAPbBr_3_)_x_ perovskite films that have been thermally annealed at various temperatures. The grain size increased with the annealing temperature. A smooth, dense perovskite film was obtained when the annealing temperature exceeded 135 °C. Lower annealing temperatures yielded rough films with some defects (or pinholes). A high-quality perovskite film is crucial in the fabrication of well-performing photovoltaic devices, as revealed by the SEM micrographs in Figure 4. Clearly, the grains merged into each other as more PbI_2_ was formed at the grain boundaries as the annealing temperature rose above 175 °C, increasing the scattering of carriers, and thereby reducing the mobility of carriers during their transportation in the perovskite film [21].

**Figure 5:**
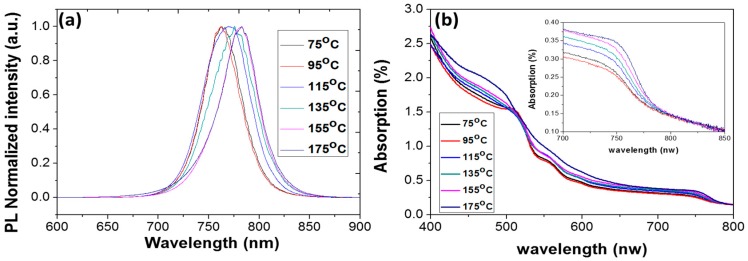
(**a**) PL and (**b**) absorbance spectra of perovskite films annealed at various temperatures.

Figure 5a displays the room-temperature PL spectra of (FAPbI_3_)_1−x_(MAPbBr_3_)_x_ that was deposited on glass substrates following annealing at various temperatures. Table 1 presents the peak positions. As the annealing temperature increases from 75 to 175 °C, the PL peak shifts from 762.1 to 782.5 nm. Figure 5b shows the absorption spectra from 400 to 800 nm of (FAPbI_3_)_1−x_(MAPbBr_3_)_x_ that was deposited on glass substrates following annealing at various temperatures. The absorption increased with the annealing temperature because the grains became larger (Figure 4). The inset presents an enlarged view of Figure 5b. The PL observations indicate that the absorption edges shift to longer wavelengths as the annealing temperature increases, owing to an offset of the stoichiometric ratio of α-FAPbI_3_ to MAPbBr_3_ since more α-FAPbI_3_ is formed and transitions to δ-FAPbI_3_ and more MAI and MABr decompose in the films. Therefore, the semiconducting band gap of the films downshifts to approaching that of the pure α-FAPbI_3_ phase. This result reveals that the composition of mixed perovskite is extremely sensitive to the temperature of annealing.

**Figure 6:**
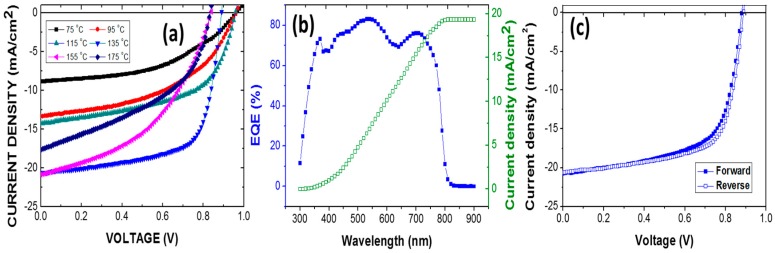
(**a**) Photocurrent J-V curves of perovskite solar cell (Ag/BCP/C_60_/(FAPbI_3_)_0.8_(MAPbBr_3_)_0.2_/PEDOT:PSS/ITO) obtained under standard 1 sun AM 1.5 simulated solar irradiation; (**b**) IPCE spectrum and (**c**) J-V curves of best device under forward and reverse scanning.

Figure 6a plots the current density as a function of the voltage (J-V) of solar cells that are based on (MAPbBr_3_)_x_(FAPbI_3_)_1−x_ and annealed at various temperatures. Table 2 presents the power conversion efficiency (Eff), short-circuit current density (J_sc_), open-circuit voltage (V_oc_) and fill factor (FF) of the (MAPbBr_3_)_x_(FAPbI_3_)_1−x_ solar cells. At annealing temperatures of less than 135 °C, the power conversion efficiency increased with the annealing temperature because J_sc_ increased as the strength of the absorption and the amount of α-FAPbI_3_ formed increased. An annealing temperature of 135 °C maximized the power conversion efficiency of the cell. The optimal device, formed by annealing at 135 °C, exhibited an outstanding performance, with J_sc_ = 20.6 mA/cm^2^, V_oc_ = 0.88 V, FF = 65.9%, and Eff = 12.0%.

The band gap of the (MAPbBr_3_)_x_(FAPbI_3_)_1−x_ film is reduced as more of the α-FAPbI_3_ phase is formed in the films (Figure 5). Therefore, annealing at 135 °C reduces the LUMO of the (MAPbBr_3_)_x_(FAPbI_3_)_1−x_ film to match that of the C_60_ layer and reduces the energy barrier to the transportation of electrons, resulting in a high FF. The value of V_oc_ is positively correlated with the difference between the HOMO of the (MAPbBr_3_)_x_(FAPbI_3_)_1−x_ film and the LUMO of the C_60_ layer (Figure 1b) [15]. Therefore, the V_oc_ following annealing at 135 °C is determined by the increase in the HOMO of the (MAPbBr_3_)_x_(FAPbI_3_)_1−x_ film. However, annealing at temperatures above 135 °C worsens the performance of the solar cell. The band gap of (MAPbBr_3_)_x_(FAPbI_3_)_1−x_ film continues to fall as the annealing temperature increases, reducing the V_oc_. Moreover, the LUMO level of (MAPbBr_3_)_x_(FAPbI_3_)_1−x_ film is lower than that of C_60_, so an energy barrier is formed, lowering the FF. As mentioned above, the PbI_2_ phase is observed, and this may block carriers, providing another cause of the low FF and the main cause of the sudden reduction of J_sc_ at an annealing temperature of 175 °C.

Figure 6b shows the IPCE of the best device and the integrated current density that is calculated from IPCE data is 19.4 mA/cm^2^. The difference between the current density from the IPCE data and that obtained from the J-V curves is only 5.8%. Therefore, the current density data are reliable. Figure 6c plots the J-V curves of the best cell under forward and reverse scanning under AM 1.5 G illumination. The efficiency difference between the forward and reverse scans is only 3.2% (Eff_forward_ = 12.0% and Eff_reverse_ = 12.4%). The hysteresis in our case is much weaker than has been reported elsewhere [16,22], indicating that high-quality (MAPbBr_3_)_x_(FAPbI_3_)_1−x_ films were formed herein by annealing.

## 4. Conclusions

In summary, the characteristics of (FAPbI_3_)_1−x_ (MAPbBr_3_)_x_ perovskite films that were thermally annealed at temperatures from 75 to 175 °C were elucidated. The optimal device that was obtained by annealing at 135 °C performed outstandingly, with short-circuit current density (J_sc_) = 20.6 mA/cm^2^, open-circuit voltage (V_oc_) = 0.88 V, fill factor (FF) = 65.9%, and power conversion efficiency (Eff) = 12.0%. The (FAPbI_3_)_1−x_(MAPbBr_3_)_x_ perovskite films must be thermally annealed to drive the interdiffusion that causes δ-FAPbI_3_ to transition into the α-FAPbI_3_ phase. PL and absorption studies reveal a shift in the band gap of the mixed perovskite following annealing. The composition of the mixed (FAPbI_3_)_1−x_(MAPbBr_3_)_x_ perovskite system is extremely sensitive to the temperature of annealing. Therefore, the annealing of mixed (FAPbI_3_)_1−x_(MAPbBr_3_)_x_ perovskite is important to control the quality of a film of that material and (ensuring OR providing) band matching in the device.

## Figures and Tables

**Table 1:** Peak positions in PL spectra of (FAPbI_3_)_1−x_(MAPbBr_3_)_x_ deposited on glass substrates and annealed various temperatures.

Thermal Annealing Temperatures	As-Deposited	75	95	115	135	155	175
PL peak (nm)	762.3	762.1	762.1	770.6	775.8	780.0	782.5

**Table 2:** Parameters of solar cells based on perovskite (FAPbI_3_)_1−x_(MAPbBr_3_)_x_ film annealed at various temperatures based on at least four devices.

Temperature (°C)	Number of Devices	V_oc_ (V)	J_sc_ (mA/cm^2^)	FF (%)	Eff (%)
75	4	1.04 ± 0.02	8.9 ± 4.4	51.7 ± 2.4	4.11 ± 0.20
95	6	0.94 ± 0.09	14.2 ± 1.9	49.4 ± 5.9	6.68 ± 0.69
115	8	0.93 ± 0.03	14.9 ± 0.6	53.6 ± 2.5	7.21 ± 0.42
135	10	0.87 ± 0.008	20.1 ± 0.5	66.4 ± 1.2	11.8 ± 0.20
155	8	0.84 ± 0.01	20.3 ± 0.6	42.9 ± 2.1	7.52 ± 0.61
175	6	0.83 ± 0.02	17.2 ± 1.0	42.0 ± 6.4	6.02 ± 0.88
